# PINK1/Parkin-mediated mitophagy is activated in cisplatin nephrotoxicity to protect against kidney injury

**DOI:** 10.1038/s41419-018-1152-2

**Published:** 2018-11-01

**Authors:** Ying Wang, Chengyuan Tang, Juan Cai, Guochun Chen, Dongshan Zhang, Zhuohua Zhang, Zheng Dong

**Affiliations:** 1 Department of Nephrology, The Second Xiangya Hospital at Central South University, Changsha, Hunan China; 2 Department of Emergency Medicine, The Second Xiangya Hospital at Central South University, Changsha, Hunan China; 3 Institute of Precision Medicine, Xiangya Hospital at Central South University, Changsha, Hunan China; 4 Department of Cellular Biology and Anatomy, Medical College of Georgia at Augusta University and Charlie Norwood VA Medical Center, Augusta, GA USA

## Abstract

Cisplatin is a widely used chemotherapeutic drug with notorious toxicity in the kidneys, which involves mitochondrial dysfunction and damage in renal tubular cells. Mitophagy is a form of selective autophagy that removes damaged or dysfunctional mitochondria to maintain cellular homeostasis. In this study, we have used mouse and cell models to examine the role and regulation of mitophagy in cisplatin nephrotoxicity. Cisplatin treatment was associated with the activation of autophagy and mitophagy. Rapamycin, a pharmacological inhibitor of mTOR, stimulated autophagy and mitophagy, and alleviated the development of cisplatin nephrotoxicity. PINK1 and Parkin were increased in kidney tissues during cisplatin treatment of mice. In PINK1 or Parkin gene knockout mouse models, both basal and cisplatin-induced mitophagy in kidneys were defective. Compared with wild-type littermates, PINK1 and Parkin knockout mice showed more severe renal functional loss, tissue damage, and apoptosis during cisplatin treatment. The results suggest that PINK1/Parkin-mediated mitophagy is activated in cisplatin nephrotoxicity and has a protective role against kidney injury.

## Introduction

Cisplatin is a widely used chemotherapy drug for the treatment of cancers of the testis, ovary, breast, lung, and other origins^1,2^. However, the use of cisplatin is limited by its side effects in normal tissues, especially it preferentially accumulates in the kidney to cause DNA damage, mitochondrial dysfunction, and various stress responses in renal tubular cells, leading to tubular cell injury, dysfunction, and even death^3–6^. The mechanism underlying cisplatin nephrotoxicity is multifactorial, involving multiple signaling pathways and molecules^3–9^. It is essential to understand these pathways to identify effective ways to alleviate cisplatin nephrotoxicity in chemotherapy.

Mitochondria, the “power plant” in cells, are the primary organelles that generate adenosine triphosphate (ATP) via oxidative phosphorylation. As such, mitochondria are critically important for the maintenance of cellular viability and activities. Damage and dysfunction of mitochondria have an important role in the pathogenesis of renal diseases, including acute kidney injury (AKI) induced by renal ischemia–reperfusion, sepsis, and nephrotoxins^10–14^. These conditions are associated with the pathological changes of mitochondrial structure and function in kidney tissues, especially in renal tubules. Mitochondrial dysfunction would then result in oxidative stress, inflammation, and cell death, inducing a rapid deterioration in renal function. This seems particular true for cisplatin-induced AKI or nephrotoxicity^3–6^.

Mitochondria-specific autophagy (mitophagy) is a fundamental process that removes excessive or damaged mitochondria selectively via autophagy, which contributes to mitochondrial quality control and cell survival^15–17^. As a selective form of autophagy, mitophagy makes use of the core machinery of autophagy for the formation of autophagosomes and autolysosomes. However, mitophagy requires a special priming process to label the mitochondria that are destined to autophagic degradation. Several pathways of mitochondrial priming have been described for mitophagy^15–17^. In this regard, the PINK1/Parkin pathway is recognized as the main pathway for mitophagy under cell stress^15–17^. Normally, PINK1 enters mitochondria to reach the mitochondrial inner membrane where it is processed by the intramembrane serine protease PARL for degradation. Interestingly, the import of PINK1 depends on mitochondrial membrane potential. And when mitochondria are depolarized in conditions such as cell stress, PINK1 accumulates on the mitochondrial outer membrane, where it recruits and phosphorylates Parkin, an E3 ubiquitin ligase. Upon phosphorylation, Parkin is activated to induce the ubiquitination of various mitochondrial outer membrane proteins^18,19^. Ubiquitin-tagged outer membrane proteins are then recognized by p62/SQSTM1 leading to encapsulation by autophagosomes and final degradation by autolysosomes^20–22^.

Mitophagy contributes critically to the pathogenesis of various diseases, such as neurodegenerative diseases, metabolic diseases, ischemia–reperfusion injury^23–25^. Our recent work demonstrated the activation and protective role of the PINK1/Parkin pathway of mitophagy in renal ischemia–reperfusion^26^. Yuan and colleagues further suggested a protective role of the PINK1/Parkin pathway during cisplatin treatment of HK2 renal tubular cells^27^. However, the role and regulation of PINK1/Parkin-mediated mitophagy in cisplatin nephrotoxicity remains largely unclear, requiring in vivo evidence to establish. Our current study was designed to investigate PINK1/Parkin-mediated mitophagy in cisplatin nephrotoxicity using both cell culture and animal models, including PINK1 and Parkin knockout (KO) mice.

## Results

### PINK1/Parkin-dependent mitophagy is induced during cisplatin treatment in BUMPT cells

**Fig. 1:**
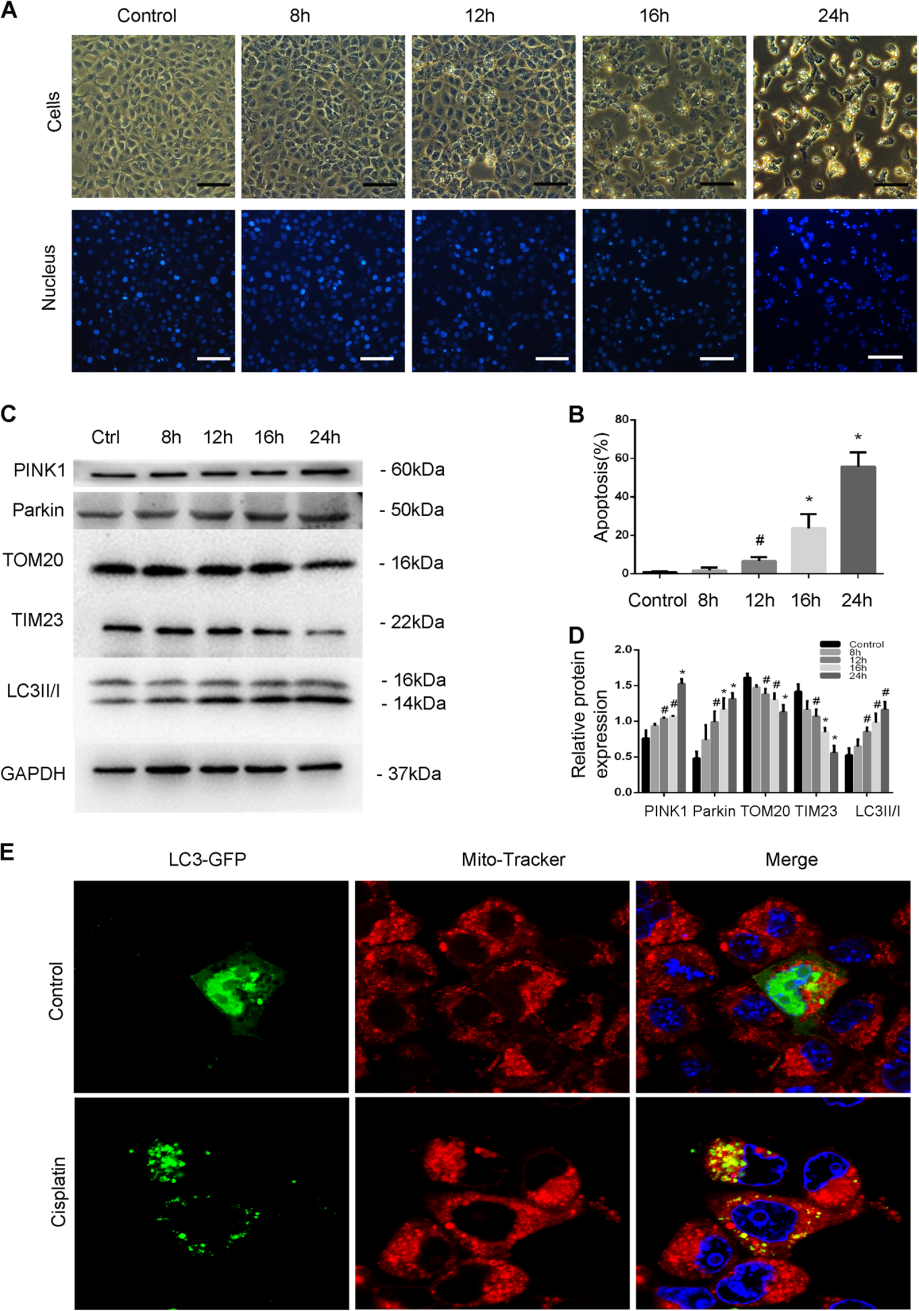
PINK1/Parkin-mediated mitophagy is induced during cisplatin treatment in BUMPT cells. BUMPT cells were treated with 20 μM cisplatin for 0–24 h. **a** Representative images of cellular and nuclear morphologies. After treatment, cells were stained with Hoechst33342 to examine by phase-contrast and fluorescence microscopy. Scale bar, 50 μm. **b** Apoptosis percentage. Apoptosis was evaluated to determine the cells with typical apoptotic morphology. **c** Whole cell lysate was collected for immunoblot analysis for PINK1, Parkin, TOM20, TIM23, LC3 II/I, and GAPDH (loading control). **d** Densitometry analysis of proteins signals on immunoblots The proteins signals were divided by GAPDH signal of the same samples to determine the ratios. **e** Co-localization of autophagosomes with mitochondria upon cisplatin treatment. BUMPT cells transiently transfected with LC3-GFP were subjected to control or cisplatin treatment. Mitochondria in these cells were then labled with MitoTracker Red FM. The cells were examined by confocal microscopy to show the co-localiztion of autophagosomes (green LC3-GFP puncta) and mitochondria (red). Scale bar: 20 μm. Data in **b** and **d** are expressed as mean ± SD. *n* = 3. ^*^P < 0.001, ^#^*P* < 0.05, significantly different from the control group

We initially examined the PINK1/Parkin-mediated pathway of mitophagy during cisplatin treatment of BUMPT cells. Cisplatin treatment led to time-dependent apoptosis as shown by cell and nuclear morphologies. Apoptosis was noticeable at 16 h and, at 24 h, many cells showed cellular shrinkage and blebbing, and nuclear condensation and fragmentation (Fig. 1a). Quantification by counting these cells revealed that cisplatin-induced 43% apoptosis in BUMPT cells for 24 h (Fig. 1b). In immunoblot analysis (Fig. 1c), cisplatin-induced LC3I to LC3 II conversion or LC3 II accumulation, indicative of the activation of autophagy. These cells also showed a reduction of the mitochondrial membrane protein TIM23 (translocase of inner mitochondrial membrane 23) and TOM20 (translocase of outer mitochondrial membrane 20 homolog), suggesting that mitochondrial clearance by mitophagy. Moreover, the expression of PINK1 and Parkin was increased, suggesting activation of PINK1-Parkin pathway of the mitophagy. Quantification of the blots by densitometry further verified the protein changes starting from 12 to 16 h and reaching high levels at 24 h of cisplatin treatment (Fig. 1d). To directly observe mitophagy, we examined the formation of “mitophagosomes” by co-localizing mitochondria and autophagosomes. BUMPT cells were transfected with LC3-GFP plasmids to reveal autophagosomes and Mito-Tracker was used to label mitochondria. The cells were subjected to cisplatin treatment or incubated in normal culture medium as control. As shown in Fig. 1e, control cells had very few GFP-LC3 puncta indicating a low level of autophagy. In sharp contrast, more LC3-GFP puncta were observed in BUMPT cells following cisplatin treatment, indicating autophagosome formation. Importantly, in cisplatin-treated cells, some LC3-GFP-labeled puncta/autophagosomes co-localized with Mito-Tracker-labeled mitochondria (Fig. 1e), indicating mitophagy. These results provided the evidence of the activation of autophagy and mitophagy during cisplatin treatment of BUMPT cells, which was accompanied by the development of apoptosis.

### Mitophagy is activated in proximal tubular cells in C57/BL6 mice

**Fig. 2:**
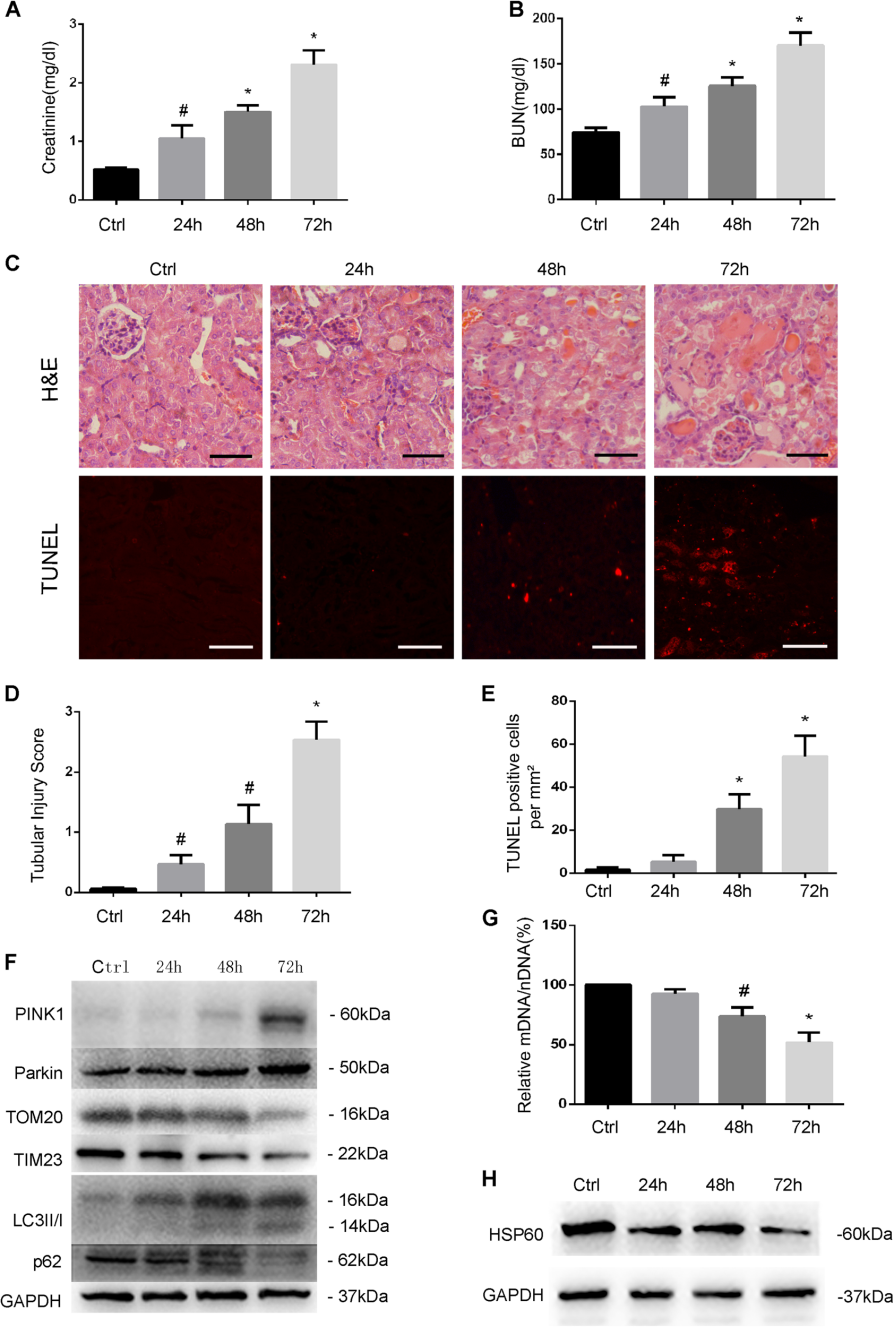
Mitophagy is induced in cisplatin-induced nephrotoxicity in mice. C57BL/6 mice (male, 8–10 weeks old) were injected with 30 mg/kg cisplatin or saline and divided into four groups for following treatments, respectively: (1) saline control; (2) cisplatin treatment for 24 h; (3) cisplatin treatment for 48 h; and (4) cisplatin treatment for 72 h. **a**, **b**. Blood samples were collected for measurements of serum creatinine (**a**) and BUN (**b**). **c** Upper panel: Representative histology of kidney cortex shown by hematoxylin and eosin (H&E) staining. Tubular injury was indicated by tubular dilation/flattening, loss of brush border, sloughing of cells into tubular lumen, formation of tubular casts, tubular degeneration, and vacuolization. Lower panel: Representative images of kidney cortex shown by TUNEL staining. Scale bar, 50 μM. **d** Tubular injury evaluated by counting the renal tubules with signs of injury. Tissue injury was scored by the percentage of damaged renal tubules (0, no damage; 1, <25%; 2, 25–50%; 3, 50–75%; and 4, >75%). **e** Quantification of TUNEL-positive cells in cisplatin treatment for 24, 48, and 72 h. **f** Relative mitochondrial DNA content (mtDNA/nDNA). **g** Immunoblot analysis of PINK1, Parkin, TOM20, TIM23, LC3II/I, p62, and GAPDH (loading control) in kidney tissues. **h** Immunoblot analysis of HSP60 and GAPDH (loading control). Data in **a**, **b** and **d**, **f** are expressed as mean ± SD, *n* = 3, ^#^*P* < 0.05, ^*^P < 0.001, significantly different from the control group

In vivo, we initially identified the occurrence of autophagy/mitophagy during cisplatin treatment in C57/BL6 mice. C57BL/6 mice (male, 8–10 weeks old) were intraperitoneally injected with 30 mg/kg cisplatin or saline as control. Blood samples were harvested to measure serum creatinine and blood urea nitrogen (BUN) to indicate the decline of renal function, and kidney tissues were collected for immunoblot/histology/apoptosis analysis at 24, 48, and 72 h. BUN and serum creatinine showed moderate increases at 24 and 48 h after cisplatin injection, and reached to 170 and 2.31 mg/dl at 72 h, respectively (Fig. 2a, b). Histological analysis following H&E staining revealed that tubular injury began to appear at 24 h and became severe at 72 h reaching tubular damage score of 2.53 (Fig. 2c, d). TdT-mediated dUTP nick-end labeling (TUNEL) assay showed that a small number of apoptosis cells began to appear at 24 h, and significant apoptosis was observed at 72 h (Fig. 2c–e). In immunoblot analysis, we detected the changes in two biochemical markers of autophagy, including the increase of LC3II and the dramatic decrease of p62 (Fig. 2f). Following cisplatin injection, the mitochondrial membrane protein TOM20 and TIM23, and the mitochondrial matrix protein HSP60 (Fig. 2f–h) were reduced, suggesting mitophagy activation. Moreover, cisplatin-induced increases in both PINK1 and Parkin (Fig. 2g). mtDNA/nDNA ratio decreased in kidney tissues of cisplatin-treated mice (Fig. 2g), further indicating the clearance of mitochondria by mitophagy. When the mtDNA/nDNA ratio in control tissue was set as 1, it decreased to 0.52 at the end of 72 h of cisplatin treatment (Fig. 2g). Together, these results indicate that the PINK1/Parkin pathway of mitophagy is activated in kidney tissues during cisplatin nephrotoxicity in vivo.

### Rapamycin attenuates cisplatin nephrotoxicity in C57BL/6 mice

**Fig. 3:**
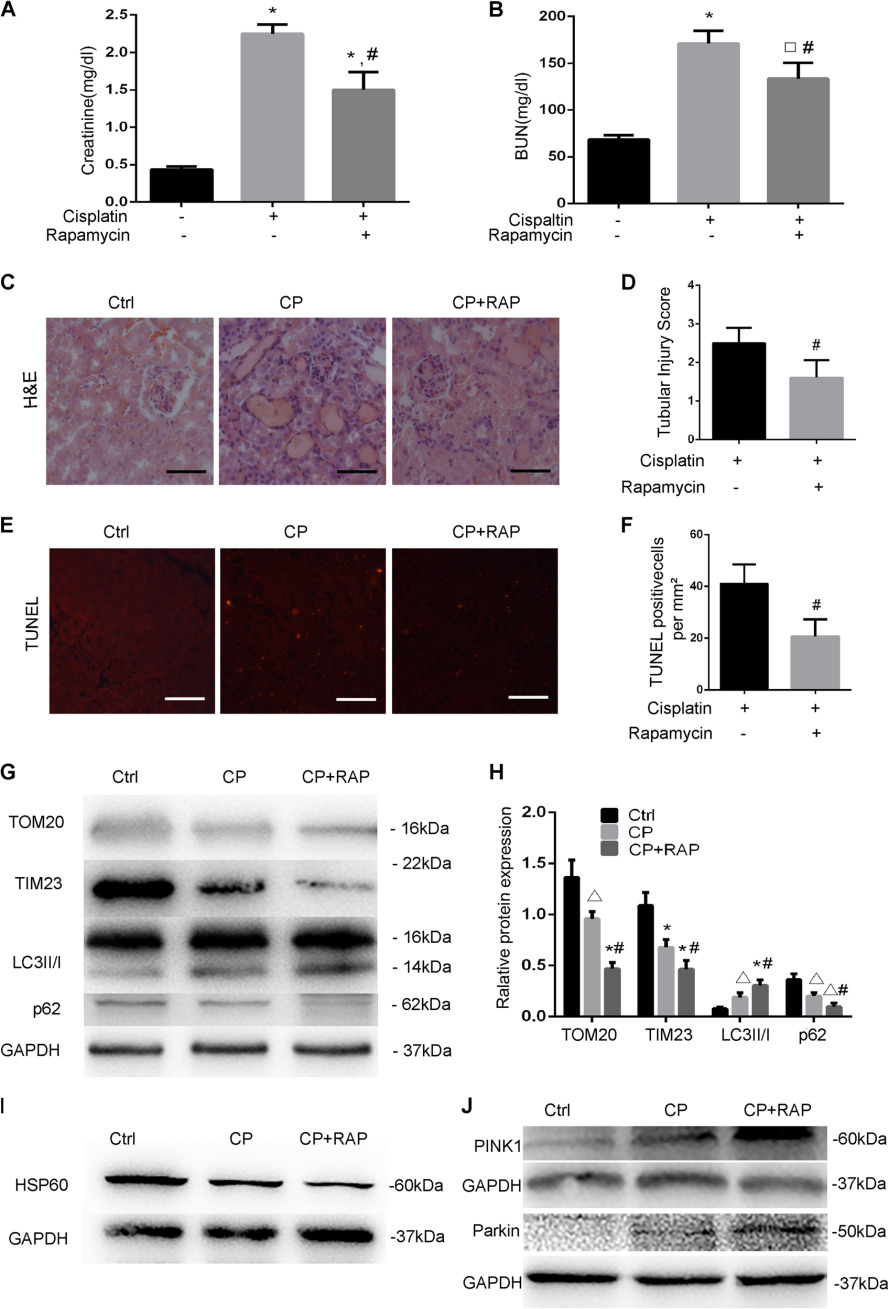
Rapamycin enhances mitophagy and protects against cisplatin-induced kidney injury in mice. C57BL/6 mice (male, 8–10 weeks old) were divided into three groups for following treatments, respectively: (1) saline control; (2) cisplatin treatment for 72 h; and (3) cisplatin + rapamycin treatment for 72 h. Cisplatin was injected at 30 mg/kg. For rapamycin, 1 mg/kg rapamycin was injected 1 h prior to and 1 day after cisplatin injection. **a**, **b** Blood samples were collected for measurements of BUN and serum creatinine. **c** Representative histology shown by H&E staining. Tubular injury was indicated by tubular dilation/flattening, loss of brush border, sloughing of cells into tubular lumen, formation of tubular casts, tubular degeneration, and vacuolization. Scale bar, 50 μM. **d** Pathological score of tubular damage in cisplatin and cisplatin + rapamycin groups. Tissue injury was scored by the percentage of damaged renal tubules (0, no damage; 1, <25%; 2, 25–50%; 3, 50–75%; and 4, >75%). **e** Representative images of TUNEL staining. Scale bar, 50 μM. **f** Quantification of TUNEL-positive cells in cisplatin and cisplatin + rapamycin groups. **g**, **i**, **j** Renal cortex tissues were analyzed for immunoblot analysis of TOM20, TIM23, LC3II/I, p62, HSP60, PINK1, Parkin, and GAPDH. **h** Densitometry analysis of proteins signals on immunoblots. The protein signals were divided by GAPDH signal of the same samples to determine the ratios. Data in **a**, **b** and **d**, **f**, **h**, **j** are expressed as mean ± SD. *n* = 3, ^*^P < 0.001, ^**^P < 0.01, ^***^P < 0.05 vs. the control group; ^#^*P* < 0.05 vs. the cisplatin group

To assess the role of mitophagy on cisplatin-induced AKI in C57BL/6 mice, we investigated the effect of rapamycin, a pharmacological inhibitor of mTOR that activates autophagy. Cisplatin-treated mice had 2.25 mg/dl serum creatinine and 171.3 mg/dl BUN, which were reduced to 1.50 and 133.6 mg/dl, respectively by rapamycin (Fig. 3a, b). Consistently, histological analysis revealed that rapamycin treatment alleviated renal tubular damage during cisplatin treatment (Fig. 3c). The tubular damage scores were 2.5 and 1.6, respectively for the cisplatin-only and the cisplatin + rapamycin groups (Fig. 3d). TUNEL assay revealed that there was significantly less tubular cell apoptosis in mice with cisplatin + rapamycin compared with those treated with cisplatin-only. To monitor mitophagy, we detected the expression of TOM20, TIM23, and HSP60 by immunoblot analysis. Cisplatin induced decreases in TOM20, TIM23, and HSP60 in renal cortical tissues, which were augmented by rapamycin (Fig. 3g–i). Rapamycin also further increased LC3-II and decreased p62 during cisplatin treatment (Fig. 3g, h). Moreover, rapamycin induced increases in both PINK1 and Parkin (Fig. 3j). Collectively, these results verified the enhancement of mitophagy and autophagy by rapamycin, which was associated with its protective effect against cisplatin nephrotoxicity.

### Mitophagy in cisplatin nephrotoxicity is attenuated in Parkin- or PINK1-KO mice

**Fig. 4:**
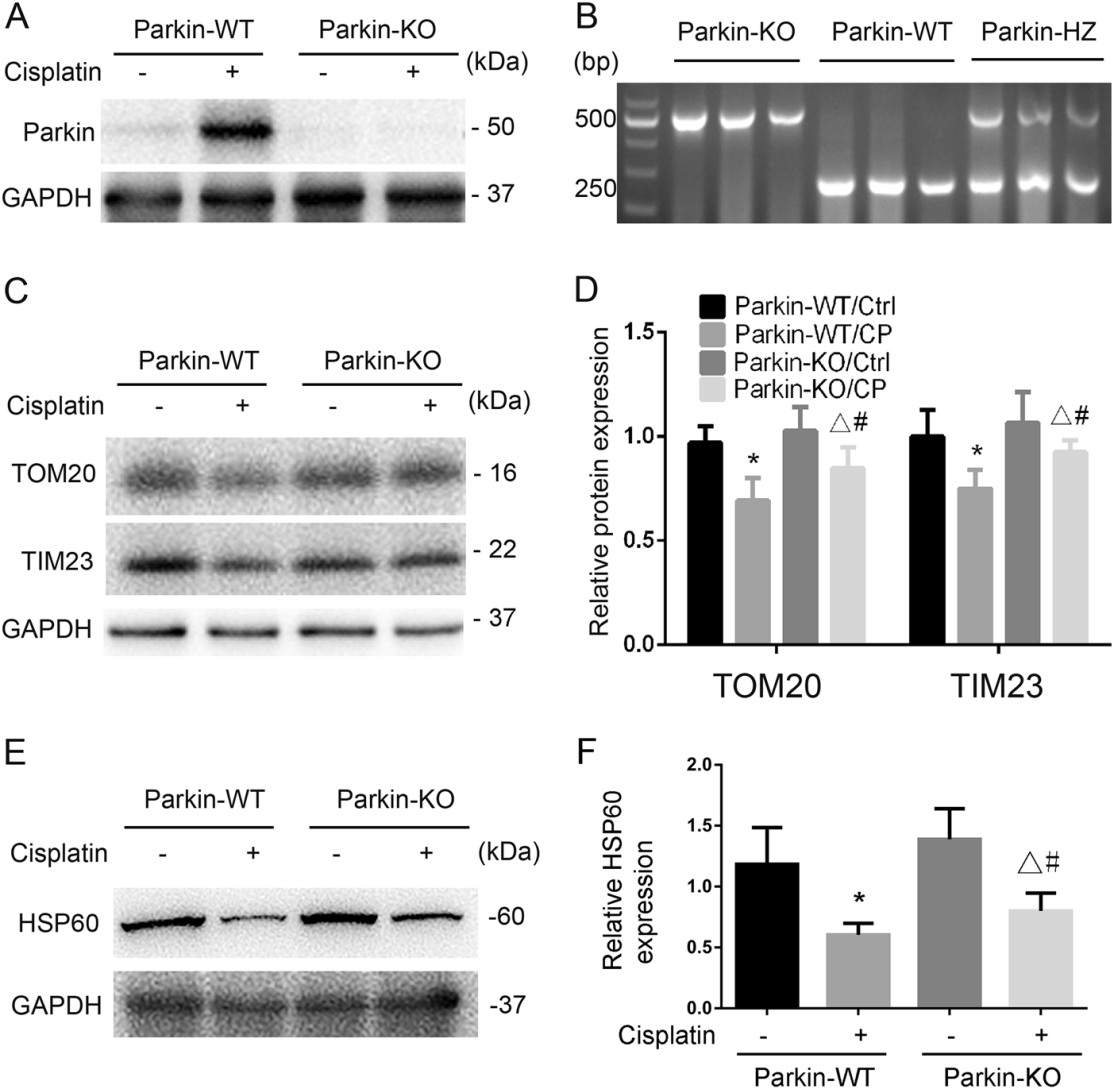
Mitophagy is suppressed in Parkin-KO mice during cisplatin nephrotoxicity. Whole tissue lysate of kidney cortex was collected from Parkin-KO and wild-type (Parkin-WT) littermate mice. **a**, **c**, **e** Immunoblot analysis of Parkin, PINK1, TOM20, TIM23, HSP60, and GAPDH. **b** Representative images of PCR-based genotyping. Genomic DNA was extracted from ear biopsy and amplified to detect wild-type (WT), Parkin KO, or Parkin heterozygote (HZ) alleles as indicated. **d**, **f** Densitometry analysis of TOM20, TIM23, and HSP60 signals on immunoblots. Mean ± SD. *n* = 4, ^*^P < 0.001 vs. the Parkin-WT control group. ^**^P < 0.05 vs. the Parkin-KO control group. ^#^*P* < 0.05 vs. Parkin-WT cisplatin group

To determine the role of PINK1/Parkin pathway of mitophagy in cisplatin-induced nephrotoxicity, we tested Parkin- or PINK1-KO mouse models. We first verified Parkin deficiency in kidney tissues of Parkin-KO mice (Fig. 4a, b). We then examined mitophagy during cisplatin treatment of wild-type (WT) and Parkin-KO mice. As shown in Fig. 4c–f, cisplatin-induced obvious decreases in TIM23, TOM20, and HSP60 in WT kidney tissues, but the decreases were significantly less in Parkin-KO mice, suggesting the involvement of PINK1/Parkin pathway in mitophagy in cisplatin nephrotoxicity.

**Fig. 5:**
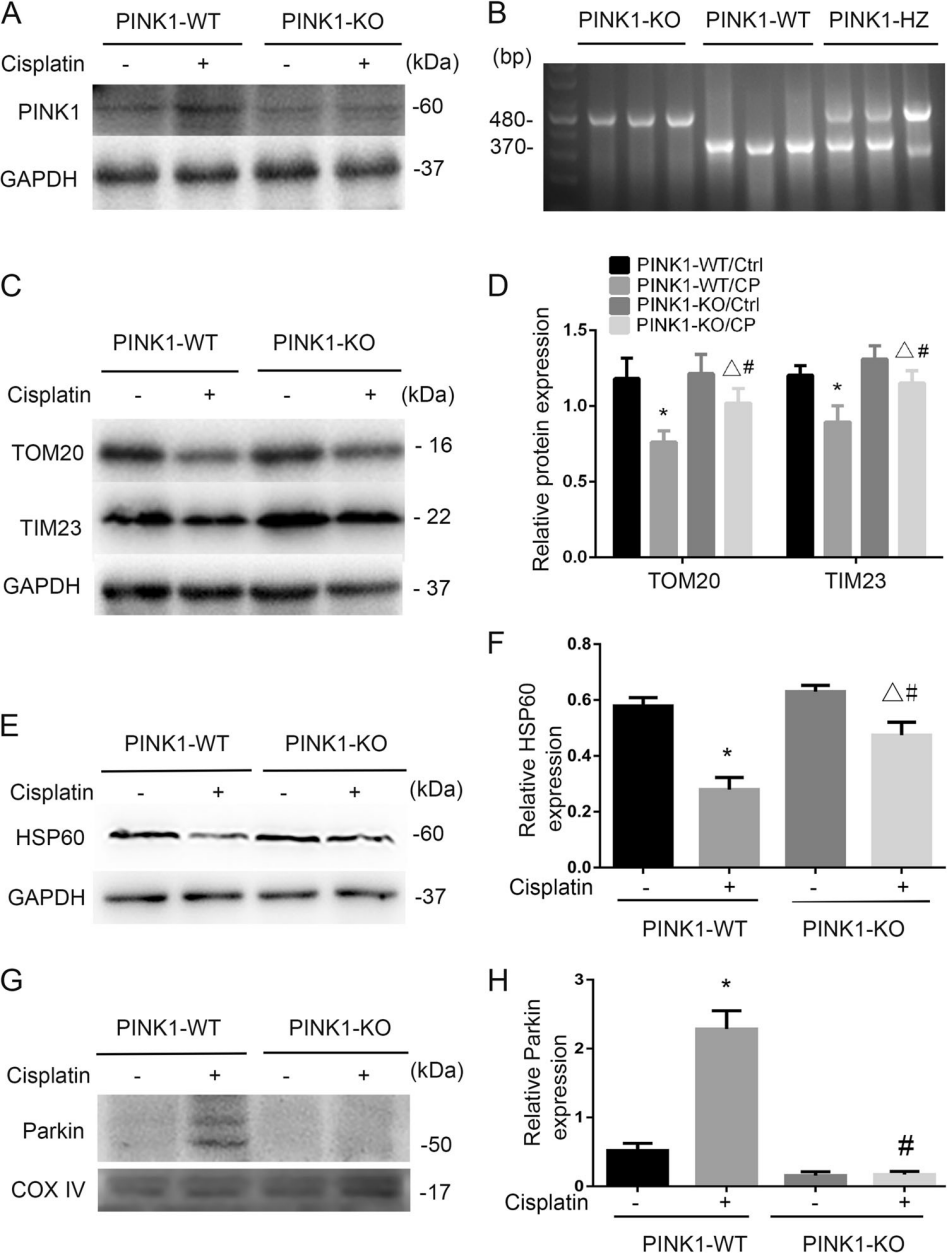
Mitophagy is inhibited in PINK1-KO mice during cisplatin nephrotoxicity. **a**, **c**, **e** Kidney cortex tissues was collected from PINK1-KO mice and PINK1-WT littermates for immunoblot analysis of Parkin, PINK1, TOM20, TIM23, HSP60, and GAPDH. **b** Representative images of PCR-based genotyping. Genomic DNA was extracted from ear biopsy and amplified to detect wild-type (WT) and PINK1-KO and PINK1 heterozygote (HZ) allele as indicated. **d**, **f** Densitometry of TOM20, TIM23, and HSP60 signals. **g**, **h** After cisplatin treatment, renal cortex was collected and extracted mitochondrial (mito) fractions followed by immunoblot analysis of Parkin, COX IV (mitochondrial marker). **g** Representative blots. **h** Densitometry of Parkin signals. Mean ± SD. *n* = 4, ^*^P < 0.001 vs. the PINK1-WT control group. ^**^P < 0.05 vs. the Parkin-KO control group. ^#^P < 0.05 vs. PINK1-WT cisplatin group

We further examined PINK1-KO mouse model. PINK1 deficiency in these mice was confirmed by genotyping and immunoblotting (Fig. 5a, b). We then compared the decreases of mitochondrial proteins TOM20, TIM23, and HSP60 induced by cisplatin in PINK1-KO and WT mice. As shown in Fig. 5c–e, cisplatin treatment reduced TOM20, TIM23, and HSP60 in WT kidney tissues, and the reduction was partially suppressed in PINK1-KO mice. Densitometry analysis further supported this conclusion (Fig. 5d–f). We further analyzed the recruitment of Parkin to mitochondria during mitophagy. To this end, mitochondria were isolated from renal cortex of cisplatin-treated or control mice, and then subjected to immunoblot analysis. As shown in Fig. 5g, h, cisplatin induced an evident Parkin recruitment to mitochondrial fraction in WT mouse kidneys, which was largely attenuated in PINK1-deficient mice. The results suggested that PINK1 contributed to mitophagy in cisplatin nephrotoxicity.

### Cisplatin nephrotoxicity is aggravated in Parkin- or PINK1-KO mice

**Fig. 6:**
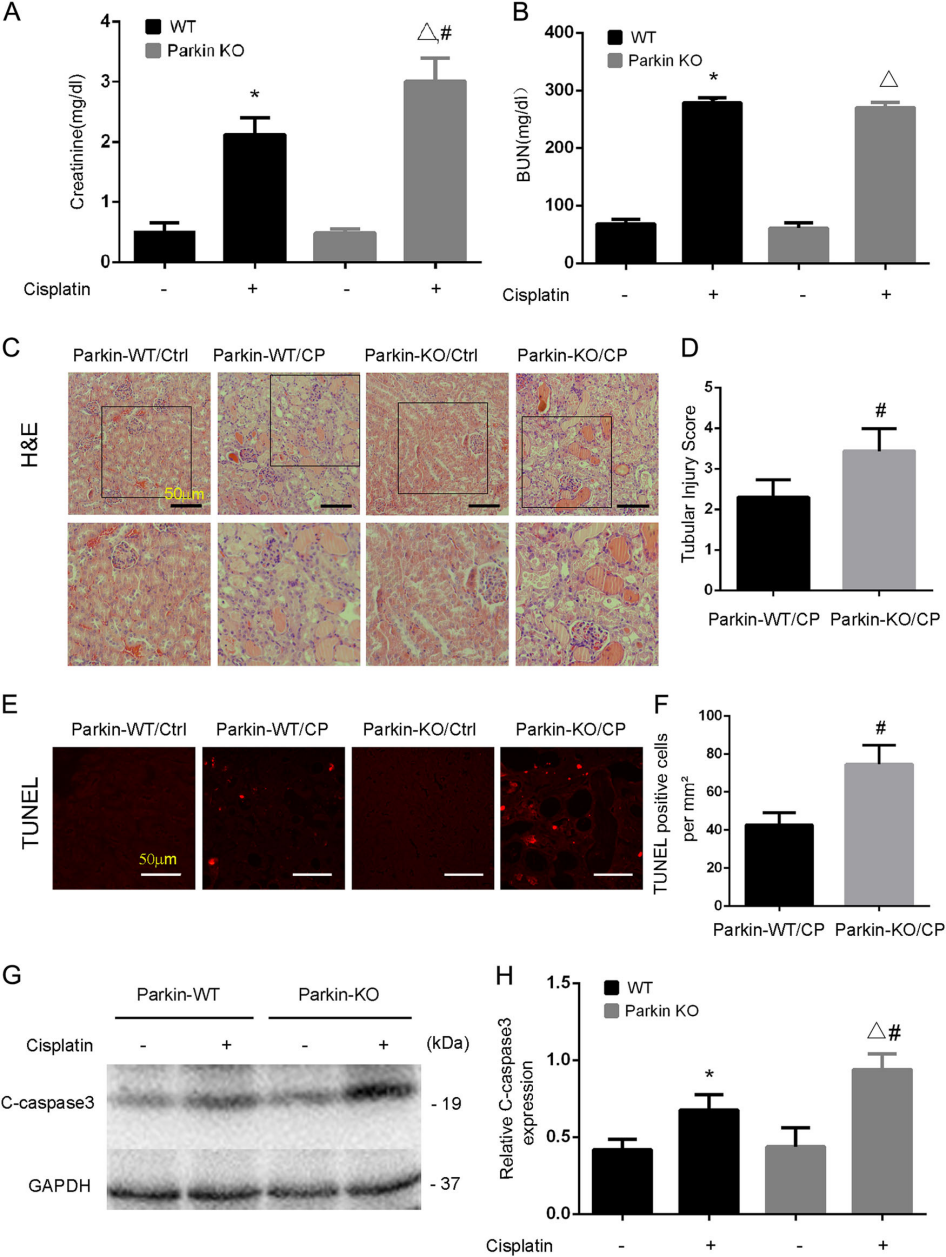
Deficiency of Parkin aggravates cisplatin-induced kidney injury. Parkin-KO mice and wide-type (WT) mice (male, 8–10 weeks) were injected with 30 mg/kg cisplatin or saline for 72 h. **a**, **b** Blood samples were collected for measurements of BUN and serum creatinine. **c** Representative histology shown by H&E staining. Tubular injury was indicated by tubular dilation/flattening, loss of brush border, sloughing of cells into tubular lumen, formation of tubular casts, tubular degeneration, and vacuolization. Scale bar, 50 μM. **d** Pathological score of tubular damage in cisplatin and cisplatin + rapamycin groups. Tissue injury was scored by the percentage of damaged renal tubules (0, no damage; 1, <25%; 2, 25–50%; 3, 50–75%; and 4, >75%). **e** Representative images of TUNEL staining. Scale bar, 50 μM. **f** Quantification of TUNEL-positive cells in cisplatin treatment for the wild-type groups and Parkin-KO groups. **g** Immunoblot analysis for cleaved caspase-3 (C-caspase 3). **h** Densitometry analysis of the cleaved-caspase 3 signals. Mean ± SD. *n* = 4, *P < 0.001 vs. the Parkin-WT control group. ^**^P < 0.001 vs. the Parkin-KO control group. ^#^*P* < 0.05 vs. Parkin-WT cisplatin group

Under unchallenged conditions, serum creatinine, and BUN were at low levels in both Parkin-KO mice and their WT littermates, indicating that renal function was normal in these mice (Fig. 6a, b). 72 h after cisplatin injection, WT mice developed acute renal failure, with serum creatinine increased to 2.12 mg/dl. In the same experiments, Parkin-KO mice had more severe loss of renal function, with serum creatinine of 3.06 mg/dl (Fig. 6a). However, there were showed similar levels of BUN in these two groups (Fig. 6b). In histological analysis following HE staining, Parkin-KO mice showed more renal tubule damage than WT mice, as indicated by tubular dilation, brush border loss, cell lysis, cast formation, and tubular degeneration (Fig. 6c). By quantification, the tubular damage score was 3.4 for Parkin-KO mice and 2.3 for WT (Fig. 6d). Further examination by TUNEL assay also showed more apoptosis in kidney tissues of Parkin-KO mice (Fig. 6e, f). Consistently, cisplatin-induced robust higher levels of caspase-3 activation in Parkin-KO kidney tissues (Fig. 6g, h).

**Fig. 7:**
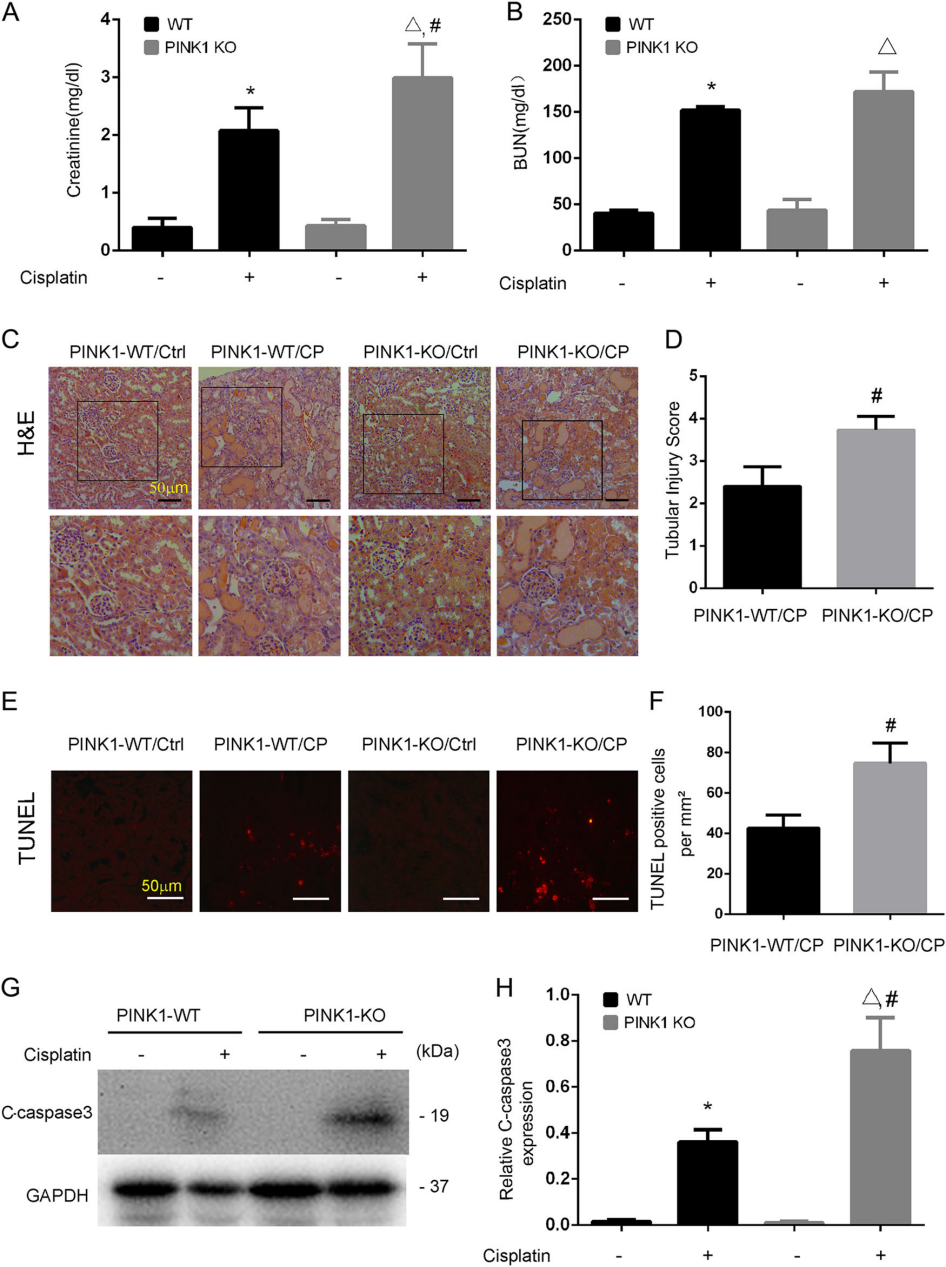
PINK1 deficiency worsens cisplatin-induced kidney injury. PINK1-KO mice and their wild-type (WT) mice (male, 8–10 weeks) were injected with 30 mg/kg cisplatin/or saline for 72 h. **a**, **b** Blood samples were collected for measurement of BUN and serum creatinine. **c**, **e** Kidney tissues were harvested for H&E staining of histology (**c**) and TUNEL assay of apoptosis (**e**). Scale bar, 50 μM. In H&E staining, yubular injury was indicated by tubular dilation/flattening, loss of brush border, sloughing of cells into tubular lumen, formation of tubular casts, tubular degeneration, and vacuolization. Scale bar, 50 μM. **d** Pathological score of tubular damage. Tissue injury was scored by the percentage of damaged renal tubules (0, no damage; 1, <25%; 2, 25–50%; 3, 50–75%; 4, >75%). **f** Quantification of TUNEL-positive cells in kidney tissues. **g** Immunoblot analysis for cleaved caspase 3. **h** Densitometry analysis of the cleaved caspase 3 signals. Mean ± SD. *n* = 4, *P < 0.001 vs. PINK1-WT control group. ^**^P < 0.001 vs. Parkin-KO control group. ^#^*P* < 0.05 vs. PINK1-WT cisplatin group

We further examined cisplatin-induced nephrotoxicity in PINK1-KO mice. As shown in Fig. 7a, PINK1-KO mice suffered more severe renal damage than WT mice after 72 h of cisplatin treatment. PINK1-KO mice had 3.00 mg/dl serum creatinine that was higher than that of WT mice (2.07 mg/dl). BUN did not significantly differ in these two groups (Fig. 7b), but histological analysis revealed that PINK1-KO mice had more widespread tubular damage as compared with WT mice (Fig. 7c). Renal tubule damage scores were 2.3 for PINK1-KO mice and 3.6 for WT mice, respectively during cisplatin treatment (Fig. 7d). In addition, TUNEL assay demonstrated that there was significant more tubular cell apoptosis after cisplatin in PINK1-KO mice than WT (Fig. 7e, f). Immunoblot analysis also showed a higher induction of cleaved/active caspase 3 in kidney tissue after cisplatin treatment in PINK1-KO mice (Fig. 7g, h).

These results indicate that cisplatin-induced nephrotoxicity is aggravated in Parkin- or PINK1-KO mice, supporting a protective role of PINK1/Parkin-mediated mitophagy in cisplatin nephrotoxicity.

## Discussion

Autophagy is activated for kidney protection in cisplatin nephrotoxicity^28–32^. However, the roles and mechanisms of mitophagy underlying cisplatin nephrotoxicity remain largely unclear. In this study, we analyzed the potential pathologic effects of mitophagy in cisplatin nephrotoxicity using both cell and animal models. Notably, this study has tested both pharmacologic modulators and genetic KO models to determine the role of mitophagy. Our results provide evidence for the activation of mitophagy in kidney tissues following cisplatin treatment. Rapamycin augmented mitophagy and afforded protection against nephrotoxic kidney injury. Notably, KO of PINK1- or Parkin-suppressed mitophagy in kidneys of cisplatin-treated mice, suggesting that PINK1/Parkin mediates the main pathway of mitophagy in nephrotoxicity. Importantly, PINK1 and Parkin KO mice showed more severe kidney injury following cisplatin treatment, supporting a protective role of PINK1/Parkin-mediated mitophagy in cisplatin nephrotoxicity.

A recent study by Yuan’s laboratory^27^ showed that in a human kidney proximal tubular HK2 cell line, knockdown of PINK1 or Parkin led to a decrease of mitophagy during cisplatin treatment, which was associated with an increase in cell injury. In contrast, overexpression of these two genes enhanced mitophagy and protected HK2 cells against cisplatin-induced cell death, providing the first evidence for a protective role of PINK1/Parkin-mediated mitophagy in cisplatin nephrotoxicity. However, it remained elusive if the PINK1/Parkin pathway was activated by cisplatin in vivo in kidneys, how much it contributed to mitophagy in cisplatin nephrotoxicity, and what role it might play in this disease condition. Our present study has addressed these important questions by using mouse models. By analyzing mitochondrial proteins and DNA, we have provided evidence of mitophagy activation in kidney tissues during cisplatin treatment (Figs. 1, 2). Pharmacological activation of autophagy by rapamycin enhanced mitophagy (Fig. 3) and protected against kidney injury. It is noteworthy that rapamycin increases autophagy in general so its effect includes, but is not limited to, mitophagy. Thus, the protective effect of rapamycin observed in our study was not solely due to mitophagy activation. Our further work showed that KO of either PINK1 or Parkin reduced mitophagy, indicating that the PINK1/Parkin pathway is the main pathway of mitophagy during cisplatin nephrotoxicity. Remarkably, KO of either PINK1 or Parkin was associated with significantly higher cisplatin nephrotoxicity in mice (Figs. 6, 7), supporting a protective role of PINK1/Parkin-mediated mitophagy in this disease condition.

In both ischemic and cisplatin nephrotoxic kidney injury, mitochondria become fragmented, contributing to renal tubular damage^33^. Mitochondrial fragmentation under these conditions is caused by the loss of mitochondrial dynamics, specifically acceleration of mitochondrial fission and arrest of mitochondrial fusion^10,34^. An important question in this research area is the fate of the fragmented mitochondria. By our observation, some fragmented mitochondria may re-fuse into long filamentous mitochondria when the insult is removed, whereas others may become irreversibly damaged, for example, by the “attacking” or insertion of Bax into mitochondrial outer membrane^35^. Our present data suggest yet another outcome, i.e., clearance of fragmented mitochondria by mitophagy. As a matter of fact, for mitophagy to occur, mitochondria need to be fragmented. In this regard, Yuan’s laboratory demonstrated that Drp-1-dependent mitochondrial fission is required for mitophagy during cisplatin treatment of HK2 cells^36^. Consistently, the latest work by Li et al.^37^ showed that mitophagy during renal ischemia–reperfusion injury depends on Drp-1-mediated mitochondrial fragmentation.

Thus far, very few studies examined mitophagy in experimental models of kidney injury. Ishihara et al.^38^ reported that Sestrin-2 and BNIP3 might regulate mitophagy in renal tubular cells under conditions of oxidative stress and hypoxia, respectively. Our recent study^26^ demonstrated a protective role of the PINK1/Parkin pathway of mitophagy in renal ischemia–reperfusion injury by using gene KO models. In nephrotoxic models, our current study and the work by Yuan’s laboratory^27,36^ also support a protective role of PINK1/Parkin-mediated mitophagy in cisplatin-induced kidney injury. Together, these studies suggest that multiple pathways of mitophagy may be activated in renal tubular cells in pathological conditions and, among them, the PINK1/Parkin pathway is a main pathway for kidney protection.

Mitophagy involves adapter or receptor proteins. In this regard, Geisler et al.^20^ reported p62 as the main receptor protein for Pink1/Parkin-mediated mitophagy. However, later studies identified several other proteins as mitophagy receptors, including TAX1BP1, NDP52, NBR1, p62, and OPTN. In 2015, Lazarou et al.^39^ conducted a comprehensive study of these known adapters and suggested that OPTN and NDP52 as the most important adapters for Pink1/Parkin-dependent mitophagy. Future research should investigate the receptor(s) for mitophagy in cisplatin-induced kidney injury and nephrotoxicity.

How does mitophagy protect kidney cells and tissues? It is generally understood that mitophagy, as a selective form of autophagy, is used by cells to degrade dysfunctional or damaged mitochondria. Cisplatin nephrotoxicity is associated with mitochondrial damage, especially that occurring in kidney tubular cells^8^. Mitochondrial damage may lead to cell death passively due to the loss of ATP production. On the other hand, it may also actively promote cell death. For example, damaged mitochondria may release apoptosis-activating factors, such as cytochrome c. In addition, damaged mitochondria may produce excessive amount of reactive oxygen species that are toxic to cells. By removing damaged mitochondria, mitophagy may reduce the level of cell stress and promote cell survival during cisplatin nephrotoxicity.

In summary, our study has demonstrated that mitophagy is activated in both cell and animal models of cisplatin nephrotoxicity. Mechanistically, mitophagy induction under this condition is mediated mainly by the PINK1/Parkin pathway. Upon activation, mitophagy protects kidneys against cisplatin toxicity likely by removing damaged mitochondria in renal tubular cells to promote cell survival.

## Methods and materials

### Cells, antibodies, and special reagents

The Boston University mouse proximal tubular (BUMPT) cell line (BUMPT-306) was initially obtained from Drs. William Lieberthal and John Shwartz at Boston University^40^. The sources of the primary antibodies in this study were as follows: anti-LC3 (12741S), anti-TOM20 (42406S), anti-Parkin (4211S), anti-GAPDH (5174S), anti-cleaved caspase-3 (9664S), HSP60 (12165P), and COX IV (4850P) from Cell Signaling Technologies; anti-TIM23 (11123-1-AP) and anti-p62 (18420-1-AP) from Proteintech; and anti-PINK1 (P0076) from Sigma-Aldrich. All secondary antibodies were from Thermo Fisher Scientific. Other reagents were purchased from Sigma-Aldrich, such as cisplatin and rapamycin. Both BUN and serum creatinine measurement kits were from BioAssay Systems.

### Animals

PINK1-KO mice and Parkin-KO mice were originally obtained from the Institute of Precision Medicine of Xiangya Hospital at Central South University (Changsha, Hunan, China), and these mouse lines were previously described^41,42^. C57BL/6 mice (8–10 weeks old, male) were purchased from SJA Laboratory Animal Corporation (Changsha, Hunan, China). Male mice of 8–12 weeks age were used in this study. All animals were housed in a pathogen-free animal facility under 12-h light/12-h dark pattern with free access to water and food. All experiments were executed in line with the protocol approved by the Institutional Animal Care and Use Committee of the Second Xiangya Hospital of Central South University.

### Cisplatin treatment of mice

Mice were injected intraperitionally with 30 mg/kg body weight cisplatin, and control mice received an intraperitional injection of the equal volume of 0.9% saline. Blood was collected for BUN and serum creatinine measurement at time 0, 24, 48, and 72 h after cisplatin treatment. Most mice were sacrificed at 72 h after cisplatin injection to collect blood samples for serum creatinine and BUN measurement, and collect renal tissues for histological and immunoblot analysis.

### Rapamycin treatment of mice

Totally, 1 mg/kg rapamycin or equal volume of 0.9% saline was injected 1 h prior to and 24 h after cisplatin (30 mg/kg) and then sacrificed at 72 h. The mice were divided into three groups for following treatment: (1) control group (injection of the equal volume 0.9% saline), (2) saline + cisplatin (injection of saline and cisplatin), and (3) rapamycin + cisplatin (injection of rapamycin and cisplatin). The method of blood harvest and kidney collection was the same as described above.

### Analysis of renal function

Renal failure or loss of renal function was revealed by serum creatinine and BUN using commercial kits as previously described^43,44^. After coagulation, blood samples were centrifuged at room temperature to collect the serum. For BUN measurement, serum samples were added to the reaction solution at room temperature for 20 min. The absorbance of 520 nm was recorded to calculate the value of BUN. For serum creatinine measurement, serum samples were added to reaction solution and the absorbance at 510 nm was recorded at 0 and 5 min of reaction. Then BUN and serum creatinine levels were calculated based on standard curves and shown as mg/dl.

### Renal histology

For histology, kidney tissues were fixed in 4% paraformaldehyde for paraffin embedding and each sample was sectioned at 4 µM for hematoxylin-eosin (H&E) staining. Histological damage was indicated by tubular dilation/flattening, loss of brush border, sloughing of cells into tubular lumen, formation of tubular casts, tubular degeneration, and vacuolization. Tissue injury was scored by the percentage of damaged renal tubules (0, no damage; 1, <25%; 2, 25–50%; 3, 50–75%; and 4, >75%.)

### Analysis of apoptosis

Apoptosis were determined in kidney tissues by TUNEL assay by using the In Situ Cell Death Detection Kit from Roche Applied Science (Indianapolis, IN) as described in our recent work^43,44^. Briefly, kidney tissues were embedded in paraffin and deparaffinized with a standard protocol. Tissue kidney sections were permeabilized with 0.1 M sodium citrate, pH 6.0 at 65 °C for 30 min and then incubated with a TUNEL reaction buffer for 1 h at 37 °C in a humidified-dark chamber. Positive staining was identified by fluorescence microscopy. Each section was selected for ten representative fields randomly and the TUNEL-positive cells per mm^2^ was counted. Apoptosis in cultured proximal tubular cells were morphological analyzed after staining with Hoechst33342. The percentage of apoptosis was evaluated according to the cells showing morphological traits including nuclear condensation and fragmentation, and formation of apoptotic bodies. Besides, apoptosis was also assessed by immunoblot analysis of cleaved caspase-3.

### Quantification of mitochondrial DNA content

Total cellular DNA of renal cortex tissue was extracted using the DNeasy Blood and Tissue kit (Qiagen, 69506) according to the manufacturer’s instruction. The relative content of mtDNA was measured as previously described^45,46^. Briefly, both mtDNA (measured by mitochondrial 16S rRNA gene) and nDNA (measured by β2-microglobulin gene) in the same samples were quantified by quantitative polymerase chain reaction (PCR) using a CFX96 Real-Time PCR Detection System (BiO-RAD) with the SyBR GreenER qPCR SuperMix (Invitrogen). The primers were: mt16S forward, 5′-ATTCCAATTCTCCAGGCATACG-3′;

mt16S reverse, 5′-GGGGTTCTTGTTTGCCGAGTT-3′;

β2-microglobulin forward, 5′-AGGGTGTGCAGAATGGGATG-3′;

β2-microglobulin reverse, 5′-GCTTCCCCCAAAGTCTACCC-3′;

GAPDH forward, 5′-AGGTCGGTGAACGGATTG-3′;

GAPDH reverse, 5′-TGTAGACCATGTAGTTGAGGTCA-3′.

### Co-localization of autophagosomes with mitochondria

BUMPT cells were transiently transfected with the LC3-GFP fusion plasmid (Addgene) by using Lipofectamine 2000 (Invitrogen) to reveal punctate autophagosome forms upon stimulation. Mitochondria in these cells were labled with MitoTracker Red FM (Molecular Probes, M22425) following the manufacturer’s instructions. The cells were examined with an Olympus FV 1000 laser-scanning confocal microscope (Olympus Corporation, Tokyo, Japan).

### Mitochondrial isolation

Mitochondria were isolated from kidneys by using mitochondrial extraction kit (Solarbio). Briefly, kidneys were rinsed in cold PBS and homogenized in ice-cold lysis buffer with a Dounce-type glass homogenizer. The homogenate was then centrifuged for three times (1000×*g* for 10 min at 4 °C) to pellet tissue debris and nuclei, yielding a supernatant enriched in mitochondria. Mitochondria from the supernatant were finally pelleted by centrifugation at 12,000×*g* and 4 °C for 10 min.

### Immunoblot analysis

Immunoblot analysis was conducted by a standard protocol^47,48^.In short, kidney cortex tissue and cell lysates were treated with 2% sodium dodecyl sulfate (SDS) buffer containing protease inhibitor cocktail (Sigma-Aldrich). Protein concentration was determined with the bicinchoninic acid reagent from Thermo Fisher Scientific. The same amounts (100 μg) of protein were loaded in each lane and resolved on SDS-polyacrylamide electrophoresis gel and then transferred to polyvinylidene diflouride membrane which was then blocked with 5% fat-free milk for 1 h before incubation with primary antibodies and horseradish peroxidaseconjugated secondary antibodies. The protein bands were revealed with a chemiluminescence kit (Termo Fisher Scientific). Densitometry of protein band signal was analyzed with Image J software (NIH) for quantification.

### Statistical analysis

Qualitative data shown in this study, including immunoblot and cell and tissue images, are representative of at least three separate experiments. Quantitative data are expressed as mean ± SD. T-test was used to determine the statistical significance between two groups. ANOVA followed by Tukey’s post-tests was conducted to identify the statistical significance in the differences among multiple groups. Statistical analysis was implemented by the GraphPad Prism software. *P* < 0.05 was considered to indicate significant differences.
